# Intersectionality in the Liminal Space: Researching Caribbean Women's Health in the UK Context

**DOI:** 10.3389/fsoc.2019.00082

**Published:** 2019-12-20

**Authors:** Nicole Andrews, Sheila Greenfield, William Drever, Sabi Redwood

**Affiliations:** ^1^Department of Health and Behavioural Sciences, Newman University, Birmingham, United Kingdom; ^2^Institute of Applied Health Research, University of Birmingham, Birmingham, United Kingdom; ^3^Hawksley Medical Practice, Birmingham, United Kingdom; ^4^School of Social and Community Medicine, University of Bristol, Bristol, United Kingdom

**Keywords:** African Caribbean women, ethnicity, intersectionality, liminality, obesity, chronic health, qualitative methodology

## Abstract

African Caribbean women in the UK who are diagnosed with chronic illnesses that are related to overweight and obesity are more likely to experience poorer outcomes than their White British counterparts. It is then important to increase understandings of how women from this ethnic group perceive health with relation to body shape and size so that interventions can be developed to prevent the development of such conditions and to improve outcomes. As such, it is important to develop research methodology that encourages participation in health research from African Caribbean women and enables the capture of in-depth data that gives insight into the nuances of health understandings related to the body and the social realities in which they exist. This paper details the methodological framework of The Big Talk project, an investigation that sought to develop a novel approach to conducting health research with seldom heard communities. The concept of intersectionality, as used in Critical Race Theory, was applied as a theoretical tool for exploring the dynamics of societal power and where this power intersects across the lived realities of race, ethnicity, gender, sexuality, (dis)ability, and class. For this research, these intersections are explored for African Caribbean women and how they relate to concepts of health, body shape, and size. This research study was conducted in spaces identified as liminal spaces for African Caribbean women: talk radio programmes; hairdressing salons and; local community groups. A Black feminist epistemological approach was used to facilitate the collection of data. The data that emerged from these liminal spaces are not necessarily definitive answers on health for African Caribbean women, but rather illuminate alternative ways of understanding the social world from the perspective of those subject to power. This phenomenon makes liminal spaces intersectional in their construction and in taking such an approach to analysis could enable finely grained details of discourses regarding health, wellness and the body to be gathered. The importance of this understanding can help to improve preventive health interventions for African Caribbean women.

## Introduction

In the UK, the risk of developing conditions such as stroke, hypertension and diabetes for African Caribbean women is higher than for White British women (Tillin et al., 2012; Conolly and Davies, 2018). The increased likelihood of the onset of such conditions for women from this ethnic group are compounded by increased risks of obesity compared to women from White British or other minority ethnic population groups (Comegna, 2017). Attempts to tackle African Caribbean women's obesity risk have involved interventions offering culturally relevant information on diet and physical activity to promote health (Mennen et al., 2001; Sharma et al., 2002; Molaodi et al., 2012). However, the complexities of health, gender, ethnicity and body shapes and sizes make this an issue that goes beyond these types of interventions.

Research conducted by Shoneye et al. (2011) with women in London found that the while the African Caribbean women had a good understanding of health risks that may be exacerbated by a larger body size, they had a more favorable opinion of a larger female body than their White British counterparts^1^. This apparent cultural normalizing of the larger female body form can be understood as existing in tension with dominant mainstream discourses promoting a slim female figure and constructing the overweight individual as problematic (Puhl and Brownell, 2001)^2^.

African Caribbean women who are diagnosed with chronic illnesses that are linked to overweight and obesity are more likely to experience poorer outcomes than their White British counterparts (Majeed-Ariss et al., 2015). Therefore, it is important to explore how African Caribbean women embody health with regard to body shape and size in order to develop interventions to improve health outcomes for this group.

The Big Talk study sought to explore issues pertaining to how issues of weight management can be initiated and explored effectively in a culturally sensitive and appropriate way with African Caribbean women by Primary Care healthcare professionals.

It sought to offer understanding into how issues of body shape and size are understood regarding health and wellbeing and how this may influence health behaviors and responses to health promotion messages. For healthcare professionals, initiating and exploring issues of weight management with patients is a complex task shaped by biomedical epistemologies (Khushf, 2013), but also influenced by mainstream understandings of health, body shape, and size. When working with diverse patient groups there is the additional complexity of varying cultural perspectives of health that gives different meaning to body shapes and sizes. This research was concerned with the discursive construction and discourses of health that African Caribbean women may refer to and how such insight could be used to improve health outcomes for this community.

This paper will detail how the concept of intersectionality, as used in CRT, was used to develop applied health research methodology with African Caribbean women for phases one and two in this study.

The term intersectionality was coined by Crenshaw and refers to “the various ways in which race and gender interact to shape the multiple dimensions of Black women's experiences” (Crenshaw, 1989, p. 139). Whilst analysis of the role of race and gender pre-dates the work of Crenshaw, use of this term names the realities of “being” in the social world as a Black women, spanning centuries of racialized and gendered existences (Hill Collins and Bilge, 2016).

The term intersectionality has its roots in Critical Race Theory (CRT). CRT suggests that for the most part, the realities of racism are shrouded by a perception of normality and it is only the most extreme and obvious forms of racism that are deemed problematic by most in society: “[b]ecause racism is an ingrained feature of our landscape, it looks ordinary and natural to persons in the culture” (Gillborn, 2015, p. 278). For CRT, there is the need to understand racial discrimination within its social, economic, and historical contexts and the ways in which marginalized groups, such as African Caribbean women, produce knowledge (Matsuda et al., 1993; Tate, 1997 As such, the particular contour of intersectionality, as posited by Crenshaw and other CRT scholars, has two strands. The first strand relates to the use of the concepts as a theoretical tool through which the process and maintenance of societal inequalities can be understood. The second strand relates to the activist element of the term which seeks to disrupt and challenge the basis of structural inequality (Gillborn, 2015, p. 279).

When applied as a prism through which nuances can be identified and analyzed, intersectionality can be used across theory, application and practice as outlined by Cho et al. (2013). This discursive application enables intersectionality to be utilized across disciplinary boundaries making it “the most important theoretical contribution that women's studies, in conjunction with related fields, has made so far” (McCall, 2005, p. 1771).

For applied health research with African Caribbean women in the UK, intersectionality facilitates the development of novel and diverse methodologies into an arena of knowledge enquiry where “disciplinary conventions import a range of assumptions and truth claims that sometime contribute to the very erasures to which intersectionality draws attention” (Cho et al., 2013, p. 793).

This paper will detail how the concept of intersectionality, as used in CRT, was used to develop applied health research methodology with African Caribbean women in the UK. The Big Talk project sought to explore the concept of health regarding body shape and size within the context of understanding how to reduce their risk of developing obesity related chronic conditions.

## The UK Context for Intersectional Applied Health Research

In understanding that the concept of intersectionality represents “multiple categories within specific socio-historical categories with specific socio-historical contexts” (Gkiouleka et al., 2018) the uniqueness of the social experience of health for African Caribbean women in the UK must provide the grounding for research methodology for applied health research. The context of UK National Health Service; race and ethnicity and; gender relations present a complex picture for the understanding of health for African Caribbean women.

The very story of the NHS from its inception to the present could not be told without acknowledging the central role that African Caribbean women have had in it and illustrates the complex relationships between diaspora, commonwealth and citizenship (Fryer, 2010). In the years following the end of World War 2, qualified and trainee healthcare professionals were recruited to work in the NHS (Kramer, 2006). As such, there has been a long visible presence of African Caribbean women in the provision of healthcare, and the NHS remains the largest employer of Black and Minority Ethnic staff in the UK (NHS, 2017). In fact, the NHS continues to recruit nurses from Caribbean counties, it is then interesting that this visible role of African Caribbean women working within the NHS can be juxtaposed with the reality that this ethnic group experience poorer health outcomes than the general population post diagnosis (Brown et al., 2007).

This hyper visibility is then juxtaposed by the relative invisibility of African Caribbean women as participants in applied health research. Under-representation in both clinical and qualitative investigations as participants have resulted in African Caribbean women being deemed to be “hard-to-reach” (Rugkasa and Canvin, 2011). However, the notion that particular groups are out of reach for researcher to engage with is disputed (Flanagan and Hancock, 2010). Use of the term “hard-to-reach” is not a neutral label; rather it is infused with preconceptions that suggest that there is something deficient about these groups (Freimuth and Mettger, 1990; Redwood et al., 2012) which hinder them from contributing to health investigations. From an intersectional perspective, the role of institutions and the knowledge that they produce has a significant impact on the ways that social inequalities are understood and perpetuate health inequalities (Hill, 2015; Kapilashrami et al., 2015).

When African Caribbean women are labeled as “hard-to-reach” in academic research, the issues of disparities in health outcomes and the under representation in health research are depicted as an intrinsic problem with African Caribbean women as a group rather than with the approach taken to engage with them. This justifies the routine exclusion of African Caribbean women from health research and provides rationale for the gaps in knowledge about understandings of health in these communities (Epstein, 2007).

When attention is paid to the interplay between the gendered realities for African Caribbean women, reasons for under representation in health research become more apparent.

The dynamics of employment (Joseph Rowntree Foundation, 2015); caring responsibilities (Office for National Statistics, 2019); experiences of racism in NHS health care as employees (Chand, 2018) and patients (Andrews et al., 2017) and; lack of trust of health research due to past histories of medical abuse of Black communities globally (Washington, 2007) give insight into under representation in applied health research. Thus, methodologies must seek to center African Caribbean women's seldom heard voices, challenging institutional notions that this is a near impossible task. Such a methodology must be rooted in a position that respects alternative ways of knowing and experiencing the world from life at the intersection.

For applied health research that works with the practical application of theory into method, the use of intersectionality as a research paradigm is complex. Choo and Ferree (2010, p. 129) suggest that one way that intersectionality can be used practically is by adopting a group centered approach, that “places the marginalized group and their perspectives at the center of the research.”

In developing this approach to methodology, the relationship between the classical theoretical notion of the sociological imagination as “the awareness of the relationship between personal experience and the wider society” (Wright-Mills, 1959 [2000]) is then re-articulated for diverse societies such as the UK through a postcolonial lens that seeks to explore these relationships through the idea of connected realities, between and within societal groups (Bhambra, 2007).

In the process of moving seldom heard voices “from margin to center” (Hooks, 2000), the methodology must allow space for seldom heard knowledges and their creation processes to be acknowledged. Further, the positioning of the research must be able to reflect personal narratives within wider social contexts for understandings that can be used to develop effective health interventions. In doing so, the research seeks to map the margins (Crenshaw, 1991) of understanding between African Caribbean women and the mainstream or biomedical perspective of health through body shape and size—research becomes the bridge between.

## A Black Feminist Epistemology

This methodology was theoretically grounded in Black feminist thought and developed using Black feminist epistemology; an approach to knowledge that recognizes uniqueness of knowledge created and affirmed within and by women in Black communities (Hill Collins, 1986, 2000). Taking such a position, seeking to place the research at the intersection with the research subjects, the research sought to offer an alternative view of how social realities of health, body shape, and size relate to African Caribbean women and; in turn how this is related to society and health service provision.

In an attempt to genuinely provide space for understandings of health, which are often very personal, using a Black feminist perspective allows subjugated knowledge to be recognized as valuable and given credence within health research.

The application of intersectionality into practice is a process that requires an interdisciplinary approach that goes beyond and builds upon the traditional qualitative health research methods whilst maintaining methodological rigor (Crosby et al., 2010). For this study, a multi-disciplinary approach incorporating understandings developed in anthropology, Black studies, cultural studies, gender studies, postcolonial studies, psychology, and media studies was used to develop a methodology that sought to empower African Caribbean women as agents of knowledge of their own health.

## The Intersectional Liminal Space

In adopting a multidisciplinary approach to encourage participation from African Caribbean women, the concept of liminality is a useful one to place in a symbiotic relationship with the notion of intersectionality. The term “liminal” was coined by the anthropologist Turner (1967, 1982) and referred to the middle stages of a ritual process during which there is a social transformation for the individual involved. For Turner, the concept of liminality was flexible beyond traditional societies and the practice of customary ceremonies and could “refer to almost anything in which there was a normally short lived period of upending of a prior hierarchy and during which power reversals occurred, or at least appeared to have occurred” (Wels et al., 2011, p. 1). As such, this makes liminality and the concept of liminal spaces a useful one beyond anthropology in other disciplines of study.

In contemporary hierarchical societies, such as the UK, spaces exist where the power relations that traditionally produce social inequalities such as racism and patriarchy are suspended and contested in forms that may be physical or metaphorical in nature. It is in such liminal, *in-between* spaces that exist between public and private where ideas can be formed, reformed, and redefined without the constraints of wider societal conventions. What emerges from the liminal space is not necessarily a definitive answer, but rather an alternative way of understanding social realities where artistic, political, cultural, and social ideas and concepts are in constant flux and contestation. For applied health research, the liminal space gives theoretical grounding for investigations to explore how marginalized people negotiate their health realties: “in short, a space where tasks and positionings become sufficiently provisional, flexible, and negotiable to enable [researchers] to weave the complexity of emerging facets of [knowledge] into a workable and productive unfolding” (Iedema et al., 2006, p. 238).

As a seldom heard group, Black women have developed sites of liminality where alternative understandings and knowledge are created and affirmed. As well as metaphorical liminal spaces, such as within the arts, they also exist in tangible form in spaces that contradict mainstream discourses of power relations and allude to different meanings.

For instance, Williams-Forson (2006) explores the relationship between food and power for Black women and suggests that the preparation of food is a liberatory act with regard to historical and contemporary social conditions and the kitchen exists as a space of emancipation from oppression. This notion opposes mainstream feminist thought that suggests that the kitchen and associated household duties are overt characteristics of patriarchal oppression (Johnson and Lloyd, 2004). Here, the importance of intersectionality as the paradigm to understand the importance is central. This example also serves to illustrate the role of liminality in what are perceived as commonplace spaces and the epistemologies developed and maintained in these spaces reflect experiences of different members of society. Research methods that can go beyond traditional boundaries of the one-to-one interview in the university or medical center setting and appreciate the dynamics of liminal spaces may yield better recruitment and responses from African Caribbean communities.

A further aim of this research study was to develop a community-based research method to increase participation from African Caribbean communities in applied health research. To capture rich accounts of understandings of health, body shape and size from a range of African Caribbean women, three methods of qualitative inquiry were used to capture natural talk using a participant observation approach in three identified liminal spaces.

The study was designed over three phases with two distinct aims. Phases one (talk radio) and two (hair dressing salons and community groups) sought to capture understandings of health through a novel community-based research method that used the concept of liminality to identify spaces for data collection. The approach to using a thematic approach for data analysis was guided by Braun and Clarke (2006). From the first cycle of thematic analysis of the data from these two phases, the contours of the understandings of health for African Caribbean women emerged. Phase three sought to explore the observed perspectives of health further by using focus groups and this data analyzed in the second cycle of thematic analysis contributed to the understanding of the wider contexts in which health may be lived and experienced by African Caribbean women (Andrews, 2017).

This project was conducted with African Caribbean communities in Birmingham, UK which has the largest African Caribbean population in England and Wales (Race Disparity Unit, 2019). In Birmingham, African Caribbean communities constitute the third largest minority ethnic group (Birmingham City Council, 2019).

Ethical procedures followed in this research investigation adhere to regulations set by University of Birmingham Code of Practice for Research (University of Birmingham, 2018). Ethical approval for this study was obtained from the University of Birmingham Ethics Board in November 2011.

As this research study sought to explore understandings of health through natural talk, the process of gaining written consent for data collection and collecting demographic details of participants would have disrupted the dynamic of data generation and impact upon the levels of participation and data quality. Ethical framework guidance published by the Economic and Social Research Council refers to instances in research where “[h]ighly formalized or bureaucratic ways of seeking consent should be avoided” (ESRC, 2015, p. 31). This is especially apt when developing novel methodologies where the process of written consent may impact on the relationship between the researcher and potential participants. As a result, the process of seeking written consent was not applied in this research and informed verbal consent from participants was sought. For phase one, using talk radio, a statement detailing the study was read to the audience before discussion took place and the public were encouraged to participate. The statement emphasized that by contacting the radio station to engage in the discussion was also consent for their data to be included in the study (BBC, 2019). For phase two a, with community groups, verbal consent took place at three intervals. Initially, consent was sought from the group co-coordinator; secondly, all potential participants were given verbal and written information about the study and verbal consent sought and; thirdly consent was confirmed before recording commenced. For phase two b, hair dressing salons, verbal consent was sought on two levels. Initially, verbal consent was sought from the potential participant after reading the participant information and; secondly, verbal consent from participants was sought immediately before data collection commenced and recorders were turned on (Andrews, 2017).

### Talk Radio as a Liminal Space

Talk radio is characterized by on-air radio discussions with programme hosts and telephone contribution from members of the audience, often on topical or controversial topics. This form of mass media is a valuable arena for research because it can be understood as one of the few spaces in society where public and private domains intersect. Audience members can choose to contribute to public discussion from their private domain by telephone or message (email or text). Over the radio, the individual is at liberty to choose whether to disclose information about themselves and their body in ways that cannot be achieved using other mass media such as television. In addition, the very nature of disembodied discussion may encourage those who have never considered taking part in health research to participate and offer views to investigations that they may have otherwise declined. As a liminal space, talk radio enables societal power dynamics based on physical appearance and relationships between researcher and participants to be temporarily suspended and re-appropriated for the duration of discussions about health and body size on-air. For African Caribbean communities, this shift is especially important as there is a recognized distrust of power relationships between African Caribbean communities and some systems of health (Brown et al., 2007; Andrews, 2017). Through this medium, those who choose to take part in the research will have more control over the process which could aid the richness of the data elicited.

Although the growth of digital technology has accelerated in the last decade, talk radio still exists as a popular and inclusive method of engaging with people for entertainment and informative purposes (Berry and Sobieraj, 2011). For African Caribbean communities in the UK, radio communication is especially important, as this form of mass media has a particular social significance (Henry, 2005, 2006). Hylton (1999) explains that because historically African Caribbeans were excluded from mainstream radio, the community developed its own network of “pirate,” or unlicensed radio stations. These community radio stations have been used for music and talk radio and are well known in the communities they serve. On-air discussion and debate have been essential elements, used to disseminate information and a space for the people to explore issues in the community. In many cities in England with significant African Caribbean communities there continues to be a strong presence of community radio stations, now more accessible via internet radio stations and/or other listening platforms.

This phase of the research was conducted using three community radio station talk shows; one local radio station that is part of a national broadcasting corporation and; one community internet radio station.

### Community Groups as Liminal Spaces

In the attempt to encourage participation in health research from groups that may be under-represented, it is also important to be mindful that research does not over-consult with particular members of the community that may be more accessible or perceived to be more receptive to taking part in research (Brackertz and Meredyth, 2009).

Historically, Black churches have been important sites for health activism (Rowland and Isaac-Savage, 2014) and have worked well with health researchers to recruit participants to explore health issues for African Caribbean communities in the UK. As such there is a tendency to approach churches to invite participation into research; this is an especially apt space for recruitment as there is continuing increase in attendance figures in Black churches (Evangelical Alliance, 2008). Whilst this is not a critique of any research that approaches church populations for health research, it must be acknowledged that the African Caribbean community is religiously diverse and sections of the community may be silenced in the process. For instance, between 2001 and 2011, UK census data reports a 0.3% increase in African Caribbeans who identify as Muslim and have a profile that is younger than the general population (Ali, 2015). Census data also shows an increase in African Caribbean Rastafarians in the UK during the same period (Office for National Statistics, 2011). There also other religions that are practiced within African Caribbean communities, including more familiar Christian denominations such as Quakers, Jehovah's Witnesses and Seventh Day Adventists, but also other religions such as Judaism and Traditional African Spirituality. There are also those who do not practice or affiliate with any particular religious orientation.

As such, investigations that seek to encourage participation from non-faith based groups that are rooted in common interests such as dress making, book clubs, parent and child groups, political activist groups, health groups, and so forth may allow for an greater representation in the research process. When considering issues of power in the research process, there are particular considerations when using community groups in this manner. The location of research data collection is a crucial element in the investigative process and to embed the research within the liminal space, it is important that the research takes place in the group's usual meeting place. As such, it is important for the research to acknowledge the importance of the physical space and appreciate that the researcher becomes an invited guest into a space that exists as a home for the group. It is important to adjust the dynamics accordingly, participant-researcher, to capture group discussion of the topic. Instead of using focus groups as a method of facilitating discussion, or group interview to ask specific questions, the discussion will be followed in its organic form, led and driven by the group. Use of this approach places the power of knowledge production with the participants and allows for group interaction to co-construct and locate understandings of health within a shared context. Importantly, as issues relating to health, wellbeing and the body are sensitive subjects, the group themselves demarcate their own boundaries of discussion and regulate the tone of the conversation that reflects the relationships within the group that cannot be assumed by the researcher to exist.

For this phase of the research a total of six community groups took part, with a total of 41 participants all over the age of 18.

### Hairdressing Salons as a Liminal Space

The third site for data collection identified as a liminal space for African Caribbean women are hairdressing salons. Whist hairdressing salons are principally rooted in economic activity, they also exist as unique locations of knowledge creation and affirmation for Black women. In the first instance, they are settings that are developed and maintained by Black women and exist within the mainstream as legitimate spaces for female beauty, yet offer services that cater to aesthetic choices that can sometimes operate outside European beauty standards. The legacy of binary distinctions between Blackness and Whiteness that emerged during the European enlightenment formed the basis of a mainstream discourse that ascribes beauty to European features and disdain for African features (Hall and Gieben, 1992); hair is one such example. As such, Black women have articulated a range of hairstyles, grooming techniques, and products that reflect, reject, or accommodate western standards. Within mainstream European society, hairstyles may be viewed as an apolitical choice, but within African Caribbean communities, the significance of a woman's hairstyle is located in a wider cultural politic and the issues of Black women's hair are a passionately debated topic within Black communities, globally and locally (Mercer, 1994; Rooks, 1996; Banks, 2000; Tharps and Byrd, 2002). Thus, the African Caribbean hairdressing salon can be read as a space that exists between mainstream and alternative discourses of femininity, sexuality, and body and beauty ideals, and discussions about such topics happen regularly. Therefore, this space is an ideal place to conduct data collection with regard research about body shape and size in the context of health.

Although the customers who may be in the salon may be from different social backgrounds (e.g. age, employment, education), they will share the commonality of engaging in body work for the purpose of body/appearance work that seeks to manage and modify “one's own looks and physical wellness” (Gimlin, 2007, p. 355). The relationship between feeling good and looking good is important in understanding the nature of excess weight and developing interventions to address obesity. Within mainstream discourse, the importance of looking good rather than improving health is the driver of many diets that regularly feature in media aimed at women. As such, distinctions between health and beauty are blurred and body work becomes the signifier of health (Monaghan, 2001). This ambiguous relationship between health and beauty is not restricted to the mainstream. In 2013, the front page of “The Voice,” the UK Black newspaper carried the headline “Do women care more about their hair than their health? (Isokariari, 2013).” The story that followed detailed the increase in overweight and obesity in Black communities in the UK and suggested that for some women the prospect of ruining their hairstyle was a barrier to physical exercise.

When exploring body work for African Caribbean women and relationships to chronic illness prevention, the hairdressing salon is an especially poignant space to conduct this research as it has been found that beauty products used by some Black women can increase susceptibility to ill health; risks that women may not always be aware of. Research has found that the products used to chemically straighten afro hair, in a process called perming, can increase the risk of pre-term birth or low birth weight for women who use perms during pregnancy (Blackmore-Prince et al., 1999; Rosenberg et al., 2005); developing uterine fibroids (Wise et al., 2012); onset of alopecia and other hair loss conditions (Khumalo et al., 2007; Olsen et al., 2011). Also, the alarming practice of skin lightening or bleaching, a chemical process that lightens the pigment of the skin has also been found to have serious health implications, such as increased susceptibility to cancers (Kooyers and Westerhof, 2006); skin diseases (Mahe et al., 2003); hypertension (Bwomda et al., 2005); and endocrine disorders, including type 2 diabetes (Olumide et al., 2008).

Additionally, the relationship between the hairdressing staff and the customer is one that contributes to the liminality of the salon and lends itself to the data collection process. There is a considerable amount of time that is spent in the hairdressing salon to achieve the desired style and; the upkeep of the style many require regular appointments at the salon. Thus, customers and hairdressers develop a particular relationship based on familiarity and trust that enable paths of communication that may not always be accessible for researchers, especially when researching sensitive topics. This relationship is one that has been used by researchers in the US, where Black hairdressers were trained as lay health advisors for Black women with particular focus on breast cancer to increase uptake of screening (Wilson et al., 2008). In the process of being granted access by the salon to conduct the research, the women may feel more comfortable to take part in the research that will be conducted as organic group discussion between customers and hairdressing staff.

In this phase of the research, a total of three hairdressing salons were used for sites of data collection. In each setting all participants were age over 18 and a total of 28 participants, including customers and salon staff contributed to the discussions.

## Lessons for Health Research at the Intersection

In using the concept of intersectionality as the basis upon which to develop methodology, this paper has shown that there is potential for applied health research to encompass both strands of the concept (Gillborn, 2015). Firstly, the methodology developed for the Big Talk project gives space for the nuances of social inequalities and the processes that uphold them to be inverted through the role of liminal spaces for African Caribbean women. The shifting of knowledge to spaces where African Caribbean women work to affirm their gender and race gives insight into how traditional paradigms of investigation contribute to re-inscribing the existing status of power dynamics that they often seek to challenge. Secondly, in the process of conducting research “with” rather than “on” African Caribbean women, this methodology seeks to give power to the seldom heard—enabling and encouraging them to be part of changing systems of knowledge production and assist in developing health services that are valuable for themselves.

For this applied health research project, the importance of African Caribbean defined and led spaces as liminal sites of knowledge is the base for CRT and the gendered element centers the women in this. For community talk radio, the importance of this as Black led public spaces where participation in discussion is rooted in the Black experience but open to all to contribute gives opportunity to observe how differing discourses of health within society relate to and within each other in this public yet disembodied forum.

As part of the Big Talk project investigation, research was conducted on three different talk radio stations that host shows that are particularly aimed at African Caribbean audiences in Birmingham. On each radio programme, the researcher was given a slot to discuss the research and invite listeners to contribute to the discussion, via telephone or other communication method supported by the radio station. During each of the talk radio programmes, callers questioned the validity of claims made by biomedical understandings of health, body shape and size, offering perspectives rooted in alternative epistemologies to counter mainstream and biomedical positions. For one radio show, the researcher was joined as a guest in the studio by a consultant physician and some callers questioned and challenged biomedical constructs of health in ways that the consultant said he had not experienced in the clinical setting and reflected a shift in traditional patient-professional relationships:

“Caller S2: I just wanted to ask, do you think that erm BMI is an accurate reading when you're going off body weight and height?” (Andrews, 2017, p. 251).

Interestingly after the show, the consultant physician expressed that he had never been challenged by patients who had such contrasting views or questions about the validity of the knowledge that shaped medical practice in such way before, a challenge that he welcomed if it could improve outcomes for patients through engagement. However, whilst there were such challenges to conventional views of health, body shape, and size whilst using this space for research, during and after each radio programme, there were many calls from listeners asking for specific health advice, so much so that announcements had to be made by the presenters that those making contact must contact healthcare professionals with regard to their issues.

Through the liminality offered by talk radio, the practical application of an intersectional lens opens this space within media up for African Caribbean women's voices that are routinely missing from mainstream areas about health. Further, this space also enabled the nuances in the perceptions of health, body shape, and size that are embodied for African Caribbean women to be given legitimate space within the discourse. After one such talk radio discussion, a woman made contact with the researcher as she felt compelled to share her experiences of living with disordered eating and the ethnic exclusions within the discourse of anorexia nervosa and bulimia that silence African Caribbean women living with such conditions and the impacts this has on their health and the lack of culturally relevant services.

In this research, the pivotal role of gender was used as the intersectional lens of understanding the knowledge of African Caribbean women through the part of the investigation based in hairdressing salons. The importance of working to gain insight into standpoint epistemologies may give applied health research findings the real potential to support change for the health outcomes of the communities it seeks to serve (Hill Collins, 1997). As spaces led and maintained by African Caribbean women, the salons are liminal spaces where knowledge of the social is co-created through dialogue centered on the women's experiences. Such co-creation of knowledge in this setting is rooted in a specific dynamic of trust, between clients and hairdresser/s and the cultivation of relationships that share gender and ethnicity.

For health research with African Caribbean women, it would not be sufficient to expect that ethnic and racial concordance would foster confidence in the researcher and the research. Rather, the trusting relationship that exists between hairdresser and client, partly rooted in a nurturing ethic of caring, a central tenet of a Black feminist epistemology (Hill Collins, 2000) is central for this phase of data collection. The roles of the hairdresser as a nurturer, performing an intimate task on the body opens up emotional boarders for sharing with others in a site that affirms the African Caribbean womanhood (in all its varying forms), though Black aesthetics. Such trust allows for the understandings at the intersection, often uncaptured by research, the opportunity to direct discussion of bodies, health, shape, and size:

“Sometimes I'm in this [hairdressing salon] chair and I talk about things so easy. I know that everyone in here will know what I mean, I don't have to mind what I say, I can just speak—it feels good. I leave with more than a hair do [laugh] I leave with peace of mind, I'm not on my own, we all feel it too. I wouldn't talk to my GP like this, I couldn't. He does know my body like you in here would” (Andrews, 2017; Andrews, Unpublished data).

The reality of the limits of trust that may exist between African Caribbean women and healthcare professionals highlights the nature of intersectionality and health for African Caribbean women. For African Caribbean women patients, feelings of isolation, judgement, and lack of understanding may impact on relationships with healthcare professionals, negatively impacting the value of discussions regarding improving health outcomes.

A central part of using an intersectional approach to developing methodology is the acknowledgment that the methods used for data collection always have the potential to further exclude some voices within the community. Whilst religious organizations in the African Caribbean community have contributed much to health activism in the UK, there are absences of some voices in these spaces for instance African Caribbean Queer voices and; those who may not practice religion in its more structured and formal sense. The role of community groups as liminal spaces within society gives opportunity to explore African Caribbean women's knowledge creation processes in sites that have developed their own group identity based on shared interest outside of formal structures.

Unlike in the hairdressing salons, women of other ethnicities were present in the group sessions and took part in the research, including women who identified as South Asian, White Irish and Black African. Whilst the research is focussed on the African Caribbean community, discourses are not produced in isolation and historically minority ethnic communities in Birmingham have lived in the same geographical areas of the city as neighbors, sharing resources and influencing each other's understandings of themselves and others. As such, the contributions from the women of other ethnic backgrounds add additional dimensions to the understandings collected, very much like the dynamics of ethnicity in the Caribbean and as told in the in histories of migration to the UK:

“Well in the Caribbean there is lots of mixing. There are lots of Indians in Trinidad and Chinese people in Jamaica” (Andrews, 2017, p. 261).

“In Ireland we weren't well off but my Mam used to fill our plates, it was like magic. It's like that for Black like Caribbean people too, plates full, pockets empty. Nobody in the area is hungry it's the way that we live. Sharing, all of us” (Andrews, 2017, p. 261).

In the group discussions of African Caribbean women, body shape and size, the hybridity of ethnicities was often explored within the context of the African diaspora and the idea that some perspectives of health were shared and much context could be taken from such links:

“In Zim [Zimbabwe] a big body is the sign of a healthy woman. Yes her body is good, she can work well, have babies and cook too the perfect woman [laugh]. I think is the same for all Black people. Well not all, but for a lot of us maybe” (Andrews, 2017, p. 261).

## Methodological Contributions

This research study sought to explore discourses of health with regard to body shape and size for African Caribbean women. The use of intersectionality as a theoretical tool allowed for the exploration of the dynamics of societal power and where this power intersects across the lived realities for the women who took part in this investigation.

Data collection was facilitated in spaces identified as liminal for African Caribbean women to encourage participation and to yield rich in-depth data. Understandings that emerge during the research process cannot claim to offer definitive answers on health for African Caribbean women, but rather illuminate alternative ways of understanding the social world from the perspective of those that are seldom heard in research.

As a characteristic of qualitative research, knowledge produced is not generalizable and it would not be accurate to posit that all African Caribbean women draw upon the discourses of health. The concept of intersectionality encourages acknowledging the diversity within groups of marginalized women. This particular aspect is of particular importance for this research as the novel method employed is also reflective of particular participants who were present and participated in those spaces at the time the research was being conducted.

A further consideration to be made of the research methodology is ethnic concordance between the researcher and the participant group. As suggested by Milner (2007, p. 395): “researchers need to reflect about themselves in relation to others—in this case, the communities and people involved in their research studies—and to acknowledge the multiple roles, identities, and positions that researchers and research participants bring to the research process”. As an African Caribbean woman conducting research with other African Caribbean women, this may have afforded the closeness that “insider” accounts can offer to further understanding and depth to analysis. However, where participants recognize and respond to the researcher's ethnicity and implied gender, they may allude to the finer detail of points making the assumption of understanding, often characterized by the phrase “you know what I mean” (Ochieng, 2010).

Whilst this academic study was conducted within a university medical school, where typically epistemologies are rooted in conventional Western frameworks of knowledge and its production (Gaventa, 1991; Ladson-Billings and Donnor, 2005), it is also shaped by personal and cultural biographies. Thus, this research is positioned between differing epistemological positions, which have influenced the methodological framework and further places importance on the lived realities of intersectionality beyond its theory.

This research study also provides direction for applied health research, illustrating that is possible to develop community-based approaches to applied health research using an intersectional perspective. It had been shown that is it possible to renegotiate boundaries of traditional research methodologies to encompass and center alternative knowledges about health in the liminal spaces in which they are developed by communities.

## Conclusion

The methodology developed for the Big Talk project shows how intersectionality can be used to mediate between the issues of and gender for African Caribbean women. This research study has shown that a multidisciplinary novel approach, using the notion of liminal spaces can be articulated into research. When used in the context of liminality, intersectionality can act as a way to understand the mediations between multiple identities and ongoing group politics that exist within wider social structures of health knowledge. For work on body shape and size, this approach to research with African Caribbean women demonstrates that there are ways that investigations can center the voices of the seldom heard and give space for them to articulate understandings that may oppose mainstream views of health. Such insights are necessary for the development of valuable and effective health intervention.

## Data Availability Statement

The datasets generated for this study are available on request to the corresponding author.

## Ethics Statement

The studies involving human participants were reviewed and approved by University of Birmingham, UK. Written informed consent for participation was not required for this study in accordance with the national legislation and the institutional requirements.

## Author Contributions

NA, SG, WD, and SR developed the initial research concept for this study and assisted with initial study design. NA undertook recruitment and data collection, managed the data, undertook initial analysis, and drafted the manuscript which was then revised critically for intellectual content by all other authors. SR, SG, and WD guided and supported the study throughout. All authors further developed the protocol, each making substantial contributions to the study's design and planning, discussed analysis and interpretation of the data in team meetings, electronically during writing up, and approved the final manuscript and agree to be accountable for its content.

### Conflict of Interest

The authors declare that the research was conducted in the absence of any commercial or financial relationships that could be construed as a potential conflict of interest.

## Footnotes

^1^These research findings also echo findings from investigations conducted with African American communities in the US that report a more favorable attitude toward the larger female body (Baptiste-Roberts et al., 2006; Gilliard et al., 2007; Schuler et al., 2008).

^2^It is important to note that while there is an observed cultural acceptance of the larger female body size and shape in African Caribbean, African American, and other Black populations, sole focus on this can silence those who are unhappy with their weight and/or are living with eating disorders (Williamson, 1998; Striegel-Moore et al., 2003).
